# Evidence of Pain, Stress, and Fear of Humans During Tail Docking and the Next Four Weeks in Piglets (*Sus scrofa domesticus*)

**DOI:** 10.3389/fvets.2019.00462

**Published:** 2019-12-11

**Authors:** Céline Tallet, Marine Rakotomahandry, Sabine Herlemont, Armelle Prunier

**Affiliations:** PEGASE, INRA, AGROCAMPUS OUEST, Saint Gilles, France

**Keywords:** ear posture, lactation, tail posture, vocalization, welfare

## Abstract

Tail docking is widely performed in pig farms to prevent tail biting. We investigated the consequences of this practice on behavioral indicators of pain and stress, and on the human-piglet relationship during lactation. Within 19 litters, piglets (1–3 days of age) were submitted on day 0 (D0) to docking with a cautery iron (D), sham-docking (S), or no docking (U). Piglets from the D and S groups were observed during the procedure (body movements and vocalizations) and just after, in isolation, during 20 s for body, tail and ear postures as well as ear movements. Piglets from the three treatments were observed in their home pen after docking on D0 and D3 afternoon for body posture, tail posture and movements. Piglets from the D and U groups were observed on D6, D12, D19, and D26 in their home pen for oral behavior, body, and tail posture. Tail damage and tear staining were scored on D5, D11, D18, and D25. A 5-min motionless human test was performed on D14. During the procedure, D piglets screamed more and with a higher intensity (*P* < 0.05) than S piglets (*n* = 48–50). Just after docking, D piglets held their ears in a posture perpendicular to the head-tail axis and changed their ear posture more often (*P* < 0.05). Between D6 and D26, D piglets kept their tail immobile (*P* < 0.001) and in a horizontal position (*P* < 0.01) more often than U piglets (*n* = 45–47). Between D11 and D25, U piglets had higher scores for tail damage and damage freshness than D piglets (0.09 < *P* < 0.02) whereas tear-stain score was similar. In the human test, D piglets interacted later with an unfamiliar human than U piglets (*P* = 0.01, *n* = 18/group). Present data indicate signs of acute pain and stress in piglets due to docking during the procedure itself and adverse consequences throughout lactation thereafter, including on their relationship with humans. On the other hand, the presence of tail lesions shows that undocked piglets are subject to more tail biting, even before weaning.

## Introduction

Tail docking is commonly performed to prevent tail biting in pigs as it reduces its prevalence 2–4 fold (1, 2). However, according to EU regulations it should not be used routinely (3). The tail is sensitive, as it is innervated (4), and there is some evidence of immediate pain and stress consequent on tail docking. Piglets struggle more during the procedure than sham operated animals (5) and they vocalize more and at higher frequencies (5–7). In the following minutes, tail wagging, tail movements, and the time spent sitting increase (5–7). Reports of the expression of pain-like behaviors (including scooting, jamming, and hunching) in the 120 min following the procedure are not consistent: some studies observed an increase in these behaviors (7) while other did not (8). The presence of neuromas detected at slaughter (4, 9, 10) suggests the existence of longer term consequences. However, only a few studies have evaluated these consequences. Most studies have focused on the tail biting consequences of not docking the tail (11) and not on the painful consequences of docking. To our knowledge, no study has attempted to observe behavioral signs of pain in the weeks following tail docking. In addition, as docking involves human intervention, human-animal relationship could be affected. Indeed, piglets may associate the negative states (fear and pain) due to docking with human presence and thus develop fear of humans, as has been observed after castration (12). The aim of the present study was to determine the consequences of tail docking on behavioral activity until weaning. We hypothesized that: (1) tail docking is a stressful and painful practice that modifies piglets' behavioral activity, tail, and ear posture shortly after the event, (2) pain and stress are maintained in the weeks following the practice and should lead to modifications of behavior including tail posture and movements or general activity, and (3) docked piglets will develop fear reactions toward humans in the weeks following the event.

## Materials and Methods

### Animals and Rearing Conditions

The experiment was performed on two batches of animals, being born either in December 2013 (9 l) or in January 2014 (10 l). All piglets in the study were born from 19 (Large White ^*^ Landrace) sows inseminated by Pietrain semen, which farrowed between Wednesday morning and Thursday evening for a given batch. Sows and their litters were reared in 1.8 m ^*^ 2.4 m farrowing pens, with crates, on plastic slatted floors with a rubber mat for piglets. Ambient temperature was set at 22°C. In addition, two infra-red lamps (on for the first week) were available to the piglets. Sows were fed with a standard lactation diet provided *ad libitum*. Sows and piglets had permanent access to fresh water. Piglets had free access to creep feed from 10 days of age. Average litter size at treatment and at weaning were 11.4 ± 1.2 and 11.3 ± 1.1 piglets, respectively (mean ± SD). In the experimental litters, a total of five experimental (2I and 3D) and seven non-experimental piglets (within 3 l) were cross fostered. Cross fostering occurred at least the day before treatment application.

### Piglets Treatments

On Thursday, experimental piglets were fitted with one colored ear tag, at random in the left or right ear, to facilitate their identification from a distance. On Friday morning (Day 0 = D0), piglets, which were then 0.5–2 days of age, were submitted within litters to one of three treatments: docked (D, *n* = 48), sham-docked (S, *n* = 50), and undocked piglets (U, *n* = 47). They were marked on their back with a special marker, so that that they could easily be identified during the behavioral observations. Whenever possible, only females were used and were equally distributed between treatments. However, due to variation in the number of females per litter, we had to include 27 male littermates. These were allocated only to the S treatment since animals from this group were observed only during the first two phases of the experiment (see Behavioral Observations), before the occurrence of surgical castration which was routinely performed in the herd at the time of the experiment. To our knowledge, there is no sex effect on pigs' behavior during the first 3 days of age. In addition, statistical analyses were performed to test for an effect of sex (27 males vs. 23 females) on the various behavioral variables within this experimental group and no statistically significant differences were detected (*P* > 0.14). Overall, each litter comprised 5–11 experimental piglets, with at least one piglet per treatment in 3 l and at least two piglets per treatment in the remaining 16 l (Supplementary Table 1).

For docking, all S and D piglets belonging to 1 l were removed simultaneously from the farrowing pen, placed in a cart bedded with fresh wood chips and transported to a separate room to undergo treatment and behavioral observations. One piglet was randomly removed from the cart by a trained handler. Immediately after removal, the treatment (S or D) was applied using a predetermined allotment established so that treatment of the first piglets of a litter was alternated and treatment of littermates was also alternated. Docking was performed with an electric-heated scissor docking iron leaving about 3 cm of the tail. This procedure took ~2 s. Sham docking was similar except that the piglet was not docked but its tail was placed on an iron bar for 2 s. Immediately after docking or sham-docking, the treated piglet was placed alone in a second partitioned cart, bedded with fresh wood chips, for 20 s of behavioral observation. Thereafter, it was moved to the other side of this second cart. The two sides of the cart were separated by a non-transparent wooden partition. Once all S and D piglets from 1 l had been treated, they were returned to the farrowing pen in the cart.

Iron injection and ear tattooing were performed on all piglets on D4 and weaning occurred at 4 weeks of age (D29).

### Behavioral Observations

The experiment was divided into four phases (Table 1):

Phase 1. During docking or sham docking, we compared the body movements and vocalizations (description in Table 2) of all 48 D and 50 S piglets. We recorded the maximum volume of the vocalizations using a sound level meter (Extech Instruments Co, USA) placed 1 m away from the head of the piglet during the process. The number of each category of vocalizations was counted by an experimenter trained to recognize the categories, which are clearly distinct. The number of body movements was counted by continuous focal sampling from video records (camcorder Sony HDR-XR200VE) with The Observer 11 (Noldus, Netherlands). The observation started once the experimenter held the piglet and the scissors, and ended when the tail was docked, or after 2 s for sham-docked piglets.
Phase 2. During the 20-s period after docking, we counted the number of each category of vocalizations via direct observations. Then, from continuous focal sampling on video recordings (camcorder Sony HDR-XR200VE), we determined the body, tail and ear postures in addition to tail movements using The Observer 11 (Noldus, Netherlands). Ear posture corresponded to the posture of the ear without an ear tag.
Phase 3. In the afternoon following docking and 3 days later (D3) we observed body and tail posture (Table 2) of 36 D, 39 S, and 37 U piglets. Piglets were marked on their back with a special marker on D0 and D2. We had to reduce the number of piglets per treatment so that observations of each litter could be done *de visu* by one observer. Piglets were selected in order to observe two piglets per experimental group and litter whenever possible. When a choice was possible, piglets were chosen at random within their litter and group. Since, in some litters, there was only one piglet per experimental group, we had to compensate by including a third piglet from the same experimental group in another litter. Litters were observed by blocks of 3 (except for one block of 4 l). Each litter of a block was observed alternately four times by 2 min scan sampling. It took ~25 min to perform this series of observations. The observer recorded body posture, tail posture, and movements of each identified piglet of the litter on a hand-held PC (PsionWorkabout, Psion PLC, London, UK) fitted with Pocket Observer 3.1. (Noldus, Netherlands). Data were transferred to The Observer XT11 (Noldus, Netherlands) to calculate the total number of observations of each posture and movement per piglet and day. In addition, tail lesions (Table 3) and tear staining (Table 4) were scored on D2.
Phase 4. Once a week (i.e., D6, D12, D19, and D26), we observed body and tail posture in addition to the oral behaviors of 45 D and 47 U piglets from the 19 l. In order to ensure that all observations were performed by a single trained observer without excessive workload, we did not continue to observe S piglets. Comparing U and D piglets allowed us to evaluate the consequences of the whole procedure including docking, handling and separation from littermates. On the day before observations, piglets were marked on their back with a special marker and tail lesions and tear staining were scored. Behavioral observations started with a scan of body and tail postures of all the piglets, followed by 5 min of focal observation of the whole litter to score all occurrences of oral behaviors and reactions performed by piglets, identifying the performer and the receiver. Once all litters had been observed, they were observed a second time in a similar manner. The observer recorded posture and activity on a hand-held PC (Psion Workabout, Psion PLC, London, UK) fitted with Pocket Observer 3.1. (Noldus, Netherlands). Data were transferred to The Observer XT11 (Noldus, Netherlands) to calculate the total number of observations of each posture and activity per animal and day of observation.

**Table 1:** Design of the experiment (U, undocked pigs; S, sham-docked pigs; D, docked pigs; M, males; F, females).

**Days**	**Husbandry event**	**Observational phase**	**Location of observations**	**Number of piglets**			**Number of sows**
				**U**	**S**	**D**	
−1, −2	Birth	No	No				19
0	Sham/tail docking	1	Husbandry table	No	27M + 23F	48F	19
0	No	2	Isolated in a cart	No	27M + 23F	48F	19
0, 3	No	3	Home pen	37F	17M + 22F	36F	19
4	Iron injection and tattoo		No	All	All	All	19
4	Tail docking	No	No	No	All	No	19
6, 12, 19, 26	No	4	Home pen	47F	No	45F	19
14	No	Human test	Test pen	18F	No	18F	18
28	Weaning	No	No	All	All	All	19

**Table 2:** Ethogram used to describe the behavioral consequences of treatments (tail docking, sham docking, and no handling/docking) in piglets.

	**Description**
**Vocalizations**	
Grunt^a^^,^^b^	Low-pitched vocalization (13)
Squeal^a^^,^^b^	High-pitched vocalization with stable frequency (13)
Scream^a^^,^^b^	High-pitched and frequency modulated vocalizations (13)
Amplitude^a^^*^	Maximum sound level during the procedure (dB).
**Body posture and movements**	
Relaxed body^a^	Piglet with the body relaxed, almost motionless
Body stiffening^a^	Piglet tense and immobile, without twisting of the spinal column
Body twisting^a^	Piglet tense and immobile with the spinal column twisted to one side. Twisting includes transitory and very brief stiffening
Forelegs movements^a^	Movements of the forelegs as if walking (pedaling in the air)
Sitting^b^^−^^d^	Piglet on its rump legs with the forelegs on the floor
Lying^b^^−^^d^	Piglet lying down
Standing^b^^−^^d^	Piglet on its four legs
Squatting^b^	Hindquarters lower than the horizontal line of the body, with the rear legs bent and not touching the floor.
**Tail posture and movements^b^^−^^d^**	
Immobile	Immobile tail, without visible movement
Moving	Visible movements
High	Higher than horizontal line
Horizontal	Horizontal line of the head-tail axis
Low to tucked	Lower than the horizontal line, or against the body or between the legs of the piglet.
**Ear posture^b^**	
Backwards	Ear directed backwards
Perpendicular	Ear perpendicular to the head-tail axis
Forwards	Ear directed forwards
Movement	Each change of posture was considered as a movement.
**Oral activities**^**d**^	
Directed to the tail	Including sniffing (contact between the snout and the tail), mouthing (taking the tail in the mouth), chewing (mouthing with movement of the jaw), biting (mouthing with a brief movement of the jaw) the tail
Directed to any other part of the body	Including sniffing, mouthing, chewing, and biting directed to the ears, the belly or any other part of the body
No reaction to oral activities	The target piglet continues its activity or inactivity
Reaction to oral activities	The target piglet changes activity by jumping, avoiding, escaping, being aggressive or vocalizing, <3 s after the start of the oral activity.

a *This was scored at docking or sham docking*.

b *This was scored for 20 s after docking or sham docking*.

c *This was scored the afternoon after docking (D0) and 3 days later (D3)*.

d *This was scored once a week till weaning*.

* *Recorded with a sound level meter (Extech Instruments Co, USA)*.

**Table 3:** Description of the various categories used for tail scoring.

**Score^a^**	**Description^a^**		**Photographs examples**
**Damage score**			
1	Intact	No damage. The skin of the tail has no marks or injuries	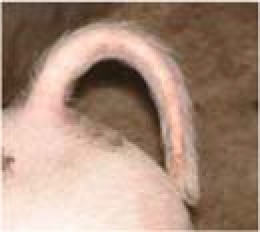
2	Swollen tail or bite marks	The tail is swollen with a red coloring or has small injuries from bites which are visible as spots/dots on the tail	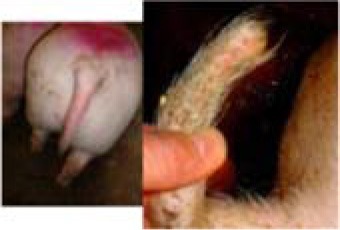
3	Open wound	The tail has one or more open wounds with puncture(s) of the skin and removal of tissue	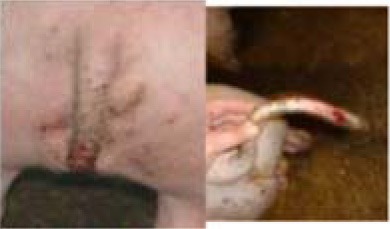
4	Swollen and open wound	The tail is swollen and has one or more open wounds with puncture(s) of the skin and removal of tissue	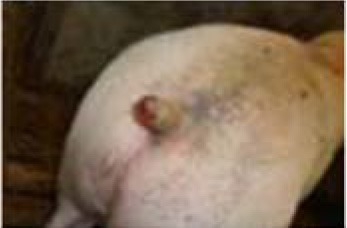
**Freshness score**			
1	No blood	No blood visible on the tail	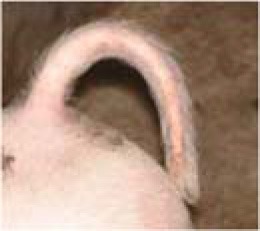
2	Dry black blood, scar	Dried blood, which is dark brown or black, is visible on the tail	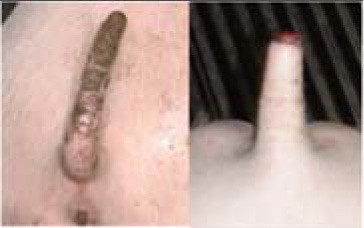
3	Dark red/brown	Somewhat dried blood is visible on the tail. It looks red to brown and feels somewhat sticky, or wet. The tail may, or may not, have a wound crust	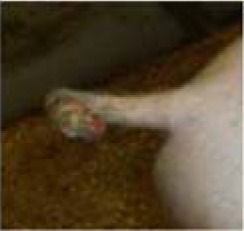
4	Red fresh blood	The tail shows fresh red blood. It feels wet due to bleeding, or exudate. The tail may or may not have a wound crust	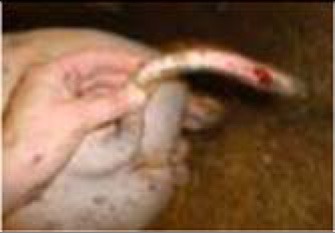

a *This scoring system was developed within the FareWellDock project, and adapted from a previous experiment (14)*.

**Table 4:** Descriptive scale used for evaluation of tear-stain scores.

**Score^a^**	**Score description^a^**
0	No signs of any staining
1	Staining is barely detectable and area stained does not extend below the eyelid
2	Staining is obvious and area stained is ~ <50% of total eye area
3	Staining is obvious and area stained is ~50–100% of total eye area
4	Staining is severe, area stained is ~≥100% of total eye area, and area stained does not extend below the mouth line
5	Staining is severe, area stained is >100% of total eye area, and area stained extends below the mouth line

a *This scoring system was developed previously (15)*.

#### Motionless Human Test

On D14, 18 D and 18 U piglets randomly selected within the 19 l were individually tested in an unfamiliar 2 m ×2 m test pen in a separate room within the rearing building. Not all piglets were evaluated in order to ensure that the test could be done in 1 day. We selected at random one D and one U piglet per litter. We had three exceptions: 1 l with 0 U and 2 D piglets, and 2 l with 1 U and 0 D piglet to compensate. The test pen was divided into 16 0.5 m ×0.5 m zones delineated by white lines drawn in chalk on the ground. A familiar experimenter carried the animal to be tested from its litter to the test pen. An unfamiliar experimenter sat motionless in the middle of one side of the pen, and the piglet was introduced at the opposite side. The test lasted for 5 min. It was video-recorded with a video camera (SONY PC25-2230P 1/3) linked to a recorder (DIVAR MR, Bosh).

The testing order was predetermined to alternate D and U piglets and litters. The observer was positioned next to the pen, not visible to the piglets. She directly recorded vocalizations (grunts and squeals) on a hand-held PC (PsionWorkabout, Psion PLC, London, UK) fitted with Pocket Observer 3.1. (Noldus, Netherlands). The latency to first movement, the latency to contact the human, the latency to look in the direction of the human, the time spent in contact, the time spent looking in the direction of the human, the time spent exploring the pen (i.e., sniffing), and the number of zones crossed (locomotor activity) were obtained from videos with The Observer XT11 (Noldus, Netherlands).

### Weight Gain

Piglets from U, S, and D treatments were individually weighed on the day following birth (D-1 or D0). Piglets from the U and D treatments were also weighed before weaning on D25. We calculated the growth rate and average daily gain of U and D piglets from these data (g/day).

### Statistics

All statistical analyses were carried out using the R software (16). Comparison of the number of animals expressing a given behavior was performed only during Phases 1 and 2. This was done using Fisher's exact tests. Other data expressed as duration or percentages of time when a pig was performing a given behavior were not normally distributed, even after log or square root transformations. Therefore, we used non-parametric statistics. During Phases 1 and 2, as well as during the motionless human test, observations of individual pigs were performed only once and were considered independent from each other. Therefore, treatments were compared with Mann Whitney U tests. During Phases 3 and 4, each experimental pig was observed more than once for each phase. Therefore, repeated measures were added to finally use only one percentage of occurrence per pig and per phase for each behavior. We also considered that observations of pigs from a litter were not independent and analyzed them using Friedman (F, comparisons between two groups) or Wilcoxon (W, comparison between three groups) tests that allowed us to use the litter as a blocking effect. When several piglets from an experimental group were present in a given litter, we added their results before performing the F or W tests so that there was only one percentage of behavioral occurrence per experimental group in each litter.

The results of the four series of tail lesion and tear staining scores were also combined by calculating for each animal the maximum value that was observed during the four series. Due to a missing value for one series, 2 l were discarded. Taking into account the low number of pigs in numerous classes of tail scores, pigs with a maximum score above 0 were regrouped into a single class. Regarding tear staining, maximum scores were either equal to 1 or 2. Therefore, all scores were analyzed as binomial data with generalized linear models using lme4, assuming a binomial error and a logit-link function. Batch and treatment were included as fixed effects. Live weight and growth rate were analyzed by analysis of variance using lme, with batch and treatment included as fixed effects and litter as a random effect. In addition, live weight at birth was added as a covariate for analyses of live weight on D25 and growth rate.

A difference was considered significant when the probability of rejecting the null hypothesis was ≤0.05. When non-parametric tests were used, values are presented as medians and interquartile range (Q25–Q75). Otherwise, percentages or adjusted mean ± SEM are presented.

## Results

### Growth

At birth, live weight was slightly lower in D than in S piglets (*P* < 0.05) whereas U piglets were intermediate (D: 1.48 ± 0.06 kg, S: 1.64 ± 0.06 kg, U: 1.55 ± 0.06 kg, *P* = 0.04). This was due to the inclusion of males in the S treatment, since the difference was no longer significant when males were omitted (D: 1.48 ± 0.06 kg, S: 1.58 ± 0.08 kg, U: 1.55 ± 0.06 kg, *P* = 0.31). On D25, S pigs were no longer included in the experiment and live weight (U: 8,036 ± 205, D: 8,211 ± 207 g) as well as daily weight gain (U: 247 ± 9 vs. D: 254. 9 g/day) did not differ between D and U treatments (*P* > 0.5). As expected, live weight at birth had a strong positive effect on daily weight gain and live weight on D25 (*P* < 0.001).

### Comparison Between Docked and Sham-Docked Piglets at Docking

Results are summarized in Table 5. During the sham-/actual docking procedure, we observed more D piglets screaming and vocalizing than S piglets, but fewer D piglets grunting than S piglets (*P* < 0.05). The total number of vocalizations and their maximum volume was also higher in D than in S piglets (*P* < 0.05). There was no difference concerning the number of piglets squealing. Body posture was also affected by the procedure; we observed more body twisting in D than in S piglets, and less relaxed body posture (*P* < 0.05). Many more D piglets moved their forelegs with twisting than S piglets (*P* < 0.05).

**Table 5:** Behavioral reactions to docking (D, *n* = 48) and sham-docking (S, *n* = 50).

	**D piglets**	**S piglets**	**P (Fisher's exact test)**	
**Number of animals expressing the vocalization category (*****and %*****)**				
Screaming	45 *(94%)*	3 *(6%)*	<0.001	
Grunting	7 *(15%)*	24 *(48%)*	<0.001	
Squealing	10 *(21%)*	17 *(34%)*	0.18	
Vocalizing	48 *(100%)*	34 *(68%)*	<0.001	
**Number of animals expressing the body posture or movement (*****and %*****)**				
Relaxed body	0 *(0%)*	28 *(56%)*	<0.001	
Body stiffening	20 *(42%)*	22 *(44%)*	0.84	
Body twisting	36 *(75%)*	4 *(8%)*	<0.001	
Forelegs movements	48 *(100%)*	17 *(34%)*	<0.001	
∙ With twisting	36 *(75%)*	4 *(8%)*	<0.001	
∙ Without twisting	12 *(25%)*	13 *(26%)*	1	
	**D piglets**	**S piglets**	**Mann-Whitney**	***P***
**Median behavior observed (and quartiles)**				
Number of vocalizations^a^	3 (2–4)	2 (1–3)	1.181	<0.001
Sound level (dB)	97 (89–103)	68 (61–73)	2.121	<0.001

a *Calculated on the animals vocalizing (i.e., 48 D and 34 S)*.

### Comparison Between Docked and Sham-Docked Piglets in Isolation Just After Docking

**Figure 1:**
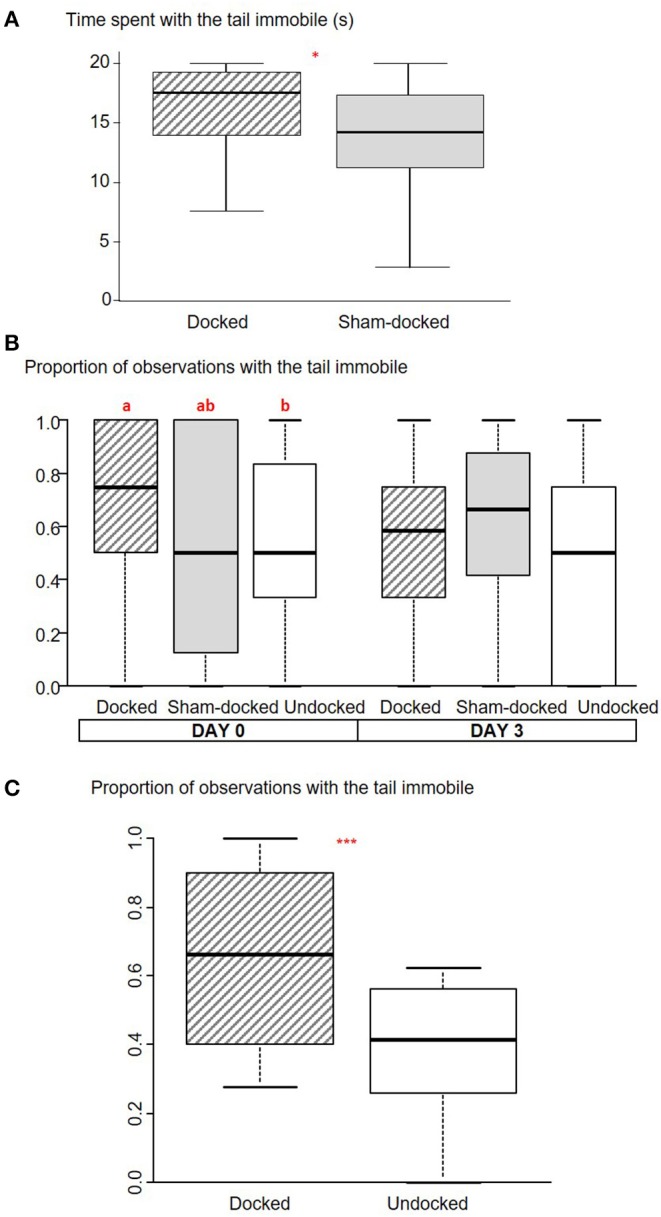
Box plot (medians and quartiles) representations of observations of the immobility of the tail at different periods for Docked, Sham-Docked, and Undocked piglets. **(A)** Time spent with the tail immobile during 20 s of observation just after docking (*n* = 48 D and 50 S: *P < 0.05). **(B)** Proportion of observations with the tail immobile on the afternoon after docking (D0) and 3 days later (D3) (*n* = 36 D, 39 S, and 37 U, a,b: *P* < 0.05). **(C)** Proportion of observations with the tail immobile for 6 scan samplings conducted over the last 3 weeks of lactation (2 scans per week) (*n* = 45 D and 47 U, ***P < 0.001).

D piglets spent more time with the tail immobile than S piglets (U = 1,540, *P* = 0.02, Figure 1A). There was no effect of docking on tail posture (*P* > 0.65); pigs from both groups spent most of their time with the tail in a low to tucked position [11.7 s (2.8–20.0), U = 1,191, *P* = 0.95] and less in a horizontal position [5.4 s (0–13.9); U = 1,262, *P* = 0.66]. Nearly all D (*n* = 41 out of 48) and S (*n* = 42 out of 50) piglets grunted, while only a few S (*n* = 3) and D (*n* = 4) squealed and none of them screamed. There was no effect of docking on the total number of vocalizations [6 (2–11), U = 1,056, *P* = 0.31]. Piglets were rarely observed sitting (5 D and 7 S) or squatting (11 D and 7 S).

**Figure 2:**
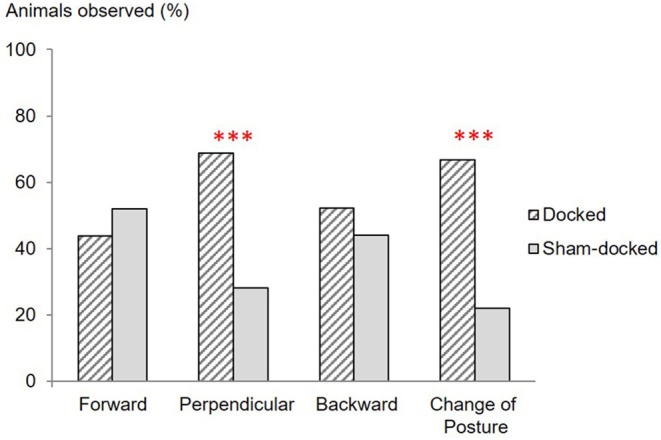
Proportions (%) of Docked (*n* = 48) and Sham-docked (*n* = 50) animals observed with their ears forward, perpendicular or backward, and observed changing their ear posture during the 20 s of observations just after docking or sham-docking. ***P < 0.001.

Ear posture differed between D and S piglets (Figure 2) with more D piglets holding their ear in a posture perpendicular to the head-tail axis and changing their ear posture (*P* < 0.05).

### Comparison Between Docked, Sham-Docked, and Undocked Piglets in Their Home Pen During the Afternoon Following Docking (D0) and 3 Days Later (D3)

On the day of docking, piglets were observed lying more often in D (quartiles: D: 75–100) than in U (quartiles: S: 60–86) and S (quartiles: 63–86) treatments (Friedman = 8.35, *P* = 0.02). U and S piglets did not differ for lying frequency. There tended to be an effect of treatment on tail immobility (Friedman = 5.6, *P* = 0.059); tails were observed as immobile more often in D piglets than in U piglets, with S piglets being intermediate (Figure 1B). There was no effect of treatment on tail posture [high: 0.17 (0.17–0.5), Friedman = 2.32, *P* = 0.31; horizontal: 0.25 (0–0.5), Friedman = 2.90, *P* = 0.24, low to tucked: 0.38 (0.13–0.67), Friedman = 1.24, *P* = 0.54]. Three days after docking (D3), there was no effect of the treatment on any of the observed variables.

### Comparison Between Docked and Undocked Piglets During Weekly Observations in Their Home Pen (D6, D12, D19, D26)

**Figure 3:**
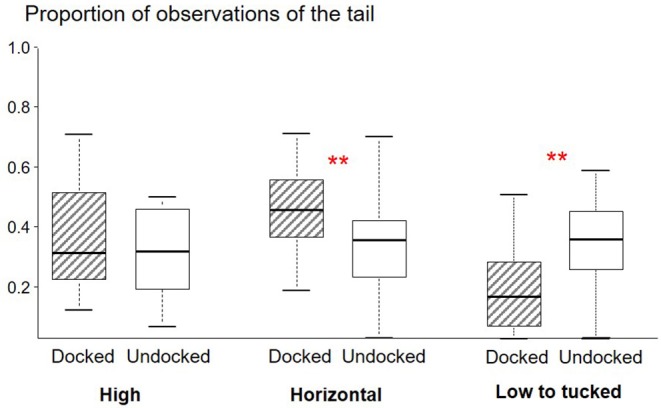
Box plot (medians and quartiles) representations of observations of the posture of the tail of Docked (*n* = 45) and Undocked (*n* = 47) piglets obtained from six scan samplings during lactation with 19 l/group; statistics are based on within litter differences. **P < 0.01.

D piglets tended to be observed lying more often than U piglets [proportion for D: 50% (41–56); U: 42% (31–50); W = 128, *P* = 0.07]. The tail of D piglets was observed as immobile more often than the tail of U piglets (W = 117, *P* < 0.001, Figure 1C). The tail of D piglets was observed in a horizontal (W = 148, *P* = 0.005) posture more often than the tail of U piglets, but less often in a low to tucked posture (W = 15, *P* = 0.001) (Figure 3).

There was no effect of docking on the total number of social oral interactions [1.22 (0.73–1.67); W = 72, *P* = 0.57] or of oral interactions directed to the tail [0.28 (0.12–0.42); W = 66, *P* = 0.65]. Following an oral interaction directed at them, D piglets reacted more often than U piglets [D: 0.78 (0.35–0.86); U: 0.44 (0.17–0.53); W = 23, *P* = 0.01].

Regarding the freshness score of the tail damage, the highest score was “4” with only one piglet on D11 reaching this score. Only one pig had a score of “3” on D11. The percentage of piglets with a freshness score above one across the four series tended to be higher in U than in D pigs (Table 6). Regarding the damage score of the tail, the highest score was “3” and only one piglet on D11 reached this score. The percentage of pigs with a damage score above 1 across the fours series of observations was significantly higher in U than in D piglets (Table 6).

**Table 6:** Tail damage and tear-stain scores in Docked and Undocked pigs.

	**Docked (*n* = 43)**	**Undocked (*n* = 43)**	***P*-value**
Number of animals with a maximum score of tail damage above 1 (and %)	2 (4.6)	8 (18.6)	0.04
Number of animals with a maximum score of damage freshness above 1 (and %)	2 (4.6)	7 (16.3)	0.07
Number of animals with a tear-stain score on one side above 1 (and %)	41 (95.3)	39 (90.7)	0.39

*Scores are combined for the four series of observations (Days 5, 11, 18, and 25)*.

The highest tear-stain score was “2.” The percentage of pigs with a tear-stain score above 1 across the fours series of observations was not significantly different between U and D piglets (Table 6).

### Comparison Between Docked and Undocked Piglets During the Motionless Human Test

**Figure 4:**
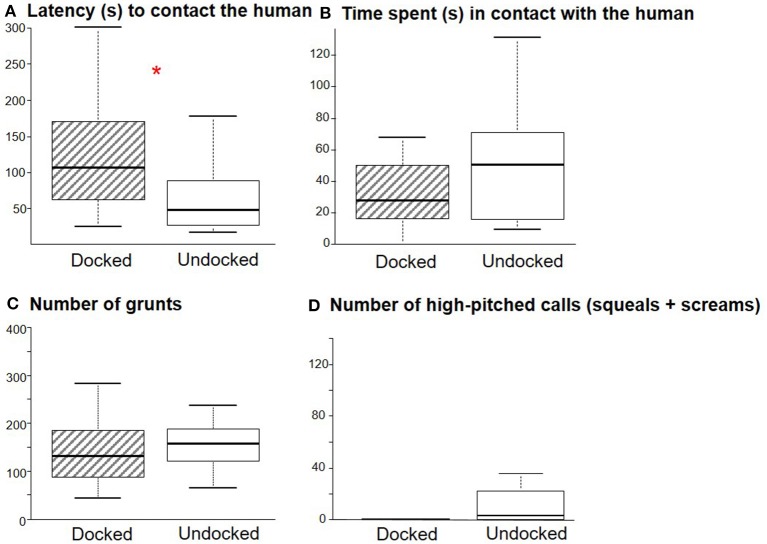
Box plot (medians and quartiles) representations of the behavior of Docked (*n* = 18) and Undocked (*n* = 18) piglets during the motionless human test. **(A)** latency (s) to contact the human, **(B)** time spent (s) in contact with the human, **(C)** number of grunts emitted, **(D)** number of high-pitched calls emitted. *P < 0.05.

It took 30 s (21–50 s) for the animals to approach the human regardless of treatment (W = 150, *P* = 0.72), but D piglets interacted later with the human than U piglets (W = 240, *P* = 0.01, Figure 4A). There was no effect of the treatment on the time spent in contact with the human (W = 113, *P* = 0.12, Figure 4B), the number of grunts (W = 123, *P* = 0.22, Figure 4C) or high-pitched vocalizations (W = 113, *P* = 0.10, Figure 4D), the number of zones crossed [98 (76–116); W = 125, *P* = 0.25], or the time spent exploring the testing pen [116 s (97–168); W = 195, *P* = 0.31].

## Discussion

When investigating the consequences of tail docking, we observed that several parameters were modified in sham-docked and intact piglets compared to docked piglets, supporting the existence of pain immediately after tail docking and in the following weeks.

### Signs of Pain and Stress During the Procedure

During docking, piglets emitted more screams and louder vocalizations than handled animals (sham-docked), in good agreement with previous data (17) which showed that the characteristics of the calls were modified by docking, i.e., mean frequencies and peak frequencies were greater in docked than in sham-docked animals. Vocalizations are expressions of emotions in mammals, and pigs in particular (18), and are modified during painful procedures like castration (19, 20). Other evidence of pain due to tail docking was found in the present experiment. Docked piglets exhibited more twisting and movements of their forelegs, and were less frequently observed in a relaxed posture. The increase in movements has previously been interpreted as a sign of attempts to escape (21). Our results therefore support previous data showing that piglets express stress and pain during the docking procedure (5, 8, 17, 22).

### Signs of Pain and Stress Immediately After the Procedure

Like preceding studies (22), we did not find any difference in body posture between docked and sham-docked piglets. However, more subtle changes were identified for the first time. Docked piglets were more often observed with their ears in a posture perpendicular to the head-tail axis or displaying ear movements. To our knowledge, the ear posture of pigs has been reported in negative situations only in one study (4), which found that the time spent with the ears in a backward posture increased after stress (social isolation combined with negative, unpredictable interventions in pigs). Backward ear posture is also a sign of negative emotion in sheep (23) and of pain in sheep and horses [lambs: (24); horses: (25)]. In our study, there was no effect of docking on the backward posture but docked piglets were more often observed with their ears in a posture perpendicular to the head-tail axis. This posture could thus be associated with pain in pigs, but this will have to be confirmed. We also observed more ear movements after docking. Ear movements have been reported to be exhibited in aversive situations in pigs (26) and sheep (23, 27). In lambs, ear movements are also associated with pain (24). Therefore, our data showing more ear movements after docking support the existence of pain. Finally, observing ear posture and movements is an efficient way to evaluate pain in pigs.

### Signs of Pain and Stress in the First Hours, Days, and Weeks After the Procedure

During the first afternoon after treatment, Docked piglets were more often observed lying than Sham and Undocked piglets, and tended to have their tail more immobile than Undocked piglets. Later on, from D6 until weaning, Docked piglets were again lying more often than Undocked piglets, and kept their tail more immobile. They also reacted more to oral contact and their tail was more often observed in a horizontal posture, but less often in a low posture. These changes could be reactions to inflammation and pain. Indeed, chronic pain is suggested to occur after docking, with the development of an increase in sensitivity of the tail and presence of neuromas (10, 28, 29). The increase in reaction to social oral contact, as well as the higher immobility of the tail, may be a protective behavior against potential interactions directed to the tail that could increase pain. Surprisingly, docked pigs exposed their tails more frequently to other pigs, since they held it more often in a horizontal and less often in a low to tucked position. This phenomenon was not related to the presence of lesions, since it was still observed when piglets with a lesion were removed from the data set (results not shown). The posture of the tail may be related to the emotional state as suggested earlier (30). Spending more time lying may be a protective reaction. Inactivity has been observed in lambs after castration and docking, and was interpreted as a way to avoid or reduce stimulation of hyperalgesic tissues (21). Alternatively, the higher frequency of lying in docked pigs may result from chronic stress related to chronic pain, since chronic stress has been shown to decrease behavioral activity and locomotion (31). In addition docking may cause infections (29) that could render animals less active.

We found more lesions on the tails of non-docked piglets, since 17% of them had a sign of lesion at least once during lactation compared to 4.4% of docked pigs. This suggests that lesions may occur even before the fattening period, which is usually considered as the risk period (32). Tail lesions at weaning were also observed in a large scale study with 480 pigs (33), in which 9% of piglets had wounds (score = 4) at weaning. Not docking the tail is thus also a source of potential stress and pain for some piglets who receive damaging acts directed toward their tail. This illustrates perfectly the dual effect of tail docking on welfare: it is a source of pain in itself but it also protects from potential pain, especially when piglets are reared in a barren environment (slatted floor, no straw bedding) which is known to be a high risk factor for tail biting (34).

The weight and growth were not influenced by docking in our experiment, in agreement with some previous reports (5, 35), whereas docking tended to decrease growth rate of piglets between 7 and 14 days old in one other study (17). Taken together, results of these experiments show that docking has no clear influence on growth of the piglets.

We did not find any effect of docking on tear staining. Tear staining (or chromodacryorrhea) seems to be a promising tool to evaluate welfare of pigs at commercial large-scale level (15) or after strong stress [isolation: (36)]. In rats, the level of chromodacryorrhea is dependent on the level of stress, as there is more secretion after high stress (maintenance work taking several hours and involving several potential stressors such as transfer of cages into a different room, noise of power tools, a brief use of an electric drill within the unit) than after mild or low stress (e.g., visits by unfamiliar humans or fighting within a cage or having a dominated social status) (37). Furthermore, the secretion is transient so that animals scored with a high level of tear staining on 1 day may be scored with no tear staining on the following day (36, 37). In our study, the lack of effect of docking on tear staining may be related to the fact that the stress caused by docking may be too short or too low.

### Consequences of Pain and Stress Associated to the Procedure on the Human-Piglet Relationship

For the first time, we showed that tail docking may have consequences on the human-animal relationship and especially on the response to an unfamiliar human. It took more time (1 min) for docked piglets to approach an unfamiliar human. Painful and stressful practices like castration or docking are potentially associated by piglets with human presence, and thus may lead piglets to fear humans. This was shown after castration (12), as castrated piglets spent less time near an unfamiliar human than entire piglets. It confirms that piglets are able to generalize their perception of humans (38). After 60–170 s, docked piglets instigated contact with the human and finally spent the same amount of time in contact with the human. The time in contact with the human remained short (<1 min) in both groups of pigs, which suggests a lack of interest in the person. It can be explained by the fact that the animals were too young to have developed a clear attraction to humans. Results may have been even more discriminative with the familiar person who handled the animals at docking and sham-docking. The effect of docking that we observed can be explained by association of the handler with pain at docking, and/or stress due to the procedure (separation, isolation, and handling). We found no evidence of an impact of docking on general stress or fear indicators (locomotion, exploration of the testing pen and vocalizations) during the test, which supports that the difference between docked and undocked pigs is due to an association between pain and the human presence rather than to an effect of chronic pain. However, this will have to be further investigated.

## Conclusion

Our study clearly shows, on the one hand, that tail docking modifies vocalizations and posture (body, tail, and ears) during the procedure and socio-oral behavior of the piglets until weaning. All these behavioral modifications are indicative of pain. On the other hand, not docking the tail renders the piglets more vulnerable to tail lesions, even before their weaning. Finally, it should be stated that pain due to tail docking involves 100% of the piglets when it is performed on a farm, without a clear possibility to reduce this pain, whereas pain due to tail biting involves only the pigs that are bitten (almost always <100%). This proportion of tail biting is highly dependent on the environment and there are many possibilities to modify the environment to make it more favorable to pig welfare and reduce tail biting risk.

## Data Availability Statement

The raw data supporting the conclusions of this article will be made available by the authors, without undue reservation, to any qualified researcher.

## Ethics Statement

This experiment was conducted at the Experimental Station of Saint-Gilles (INRA, France, GPS: 48.1452,−1.830114). All procedures were reviewed and approved by the French Ministry of Agriculture under the number 01491.03 in accordance with the European legislation and the French rural and sea fishing code's articles R.214-87 to R.214-126.

## Author Contributions

CT and AP conceived and designed the experiment. MR, CT, and SH conducted the study. MR, CT, AP, and SH analyzed the data. The manuscript was prepared and edited by CT and AP.

### Conflict of Interest

The authors declare that the research was conducted in the absence of any commercial or financial relationships that could be construed as a potential conflict of interest.
