# Supplementary material for: Evidence of Pain, Stress, and Fear of Humans During Tail Docking and the Next Four Weeks in Piglets (Sus scrofa domesticus)
Source: Front Vet Sci. 2019 Dec 11;6:462. doi: 10.3389/fvets.2019.00462 (PMC6917581; doi:10.3389/fvets.2019.00462)
Supplement: Supplementary file 1 [file Table_1.DOCX]

|  |  |  | Number of piglets for phase 3 | | | Number of piglets for phase 4 | | Number of piglets for the human test | | Number of piglets for tail and TS scores | | Littersize | | |
| --- | --- | --- | --- | --- | --- | --- | --- | --- | --- | --- | --- | --- | --- | --- |
| Batch | Farrowing pen | Sow number | Docked | Undocked | Sham-docked | Docked | Undocked | Docked | Undocked | Docked | Undocked | at treatment | | at weaning |
| 1 | 1 | 832283 | 2 | 2 | 2 | 2 | 2 | 1 | 1 | 2 | 2 | 12 | 12 | |
| 1 | 2 | 241176 | 2 | 2 | 2 | 3 | 3 | 1 | 1 | 3 | 3 | 12 | 12 | |
| 1 | 3 | 230313 | 2 | 2 | 2 | 2 | 2 | 1 | 1 | 2 | 2 | 11 | 11 | |
| 1 | 5 | 241085 | 2 | 2 | 2 | 4 | 4 | 1 | 1 | 4 | 4 | 12 | 12 | |
| 1 | 8 | 141809 | 2 | 2 | 2 | 3 | 3 | 1 | 1 | 3 | 3 | 10 | 10 | |
| 1 | 9 | 1230312 | 2 | 2 | 2 | 3 | 2 | 1 | 1 | 3 | 2 | 11 | 11 | |
| 1 | 10 | 231155 | 2 | 2 | 2 | 2 | 2 | 1 | 1 | 2 | 2 | 12 | 12 | |
| 1 | 11 | 230019 | 2 | 2 | 2 | 3 | 2 | 1 | 1 | 3 | 2 | 11 | 11 | |
| 1 | 12 | 231154 | 2 | 2 | 2 | 3 | 3 | 1 | 1 | 3 | 3 | 13 | 11 | |
| 2 | 1 | 230512 | 2 | 2 | 2 | 4 | 4 | 1 | 1 | 4 | 3 | 12 | 12 | |
| 2 | 2 | 221475 | 2 | 2 | 2 | 2 | 2 | 1 | 1 | 2 | 2 | 10 | 10 | |
| 2 | 3 | 341025 | 2 | 2 | 2 | 3 | 3 | 1 | 1 | 2 | 3 | 12 | 12 | |
| 2 | 4 | 32388 | 1 | 3 | 2 | 2 | 3 | 1 | 1 | 2 | 2 | 11 | 11 | |
| 2 | 6 | 241797 | 1 | 1 | 3 | 1 | 1 | 1 | 1 | 1 | 1 | 11 | 11 | |
| 2 | 7 | 241833 | 1 | 2 | 3 | 1 | 2 | 1 | 1 |  |  | 11 | 11 | |
| 2 | 8 | 341026 | 2 | 2 | 2 | 2 | 3 | 1 | 1 | 2 | 3 | 13 | 13 | |
| 2 | 9 | 832348 | 2 | 1 | 2 | 0 | 0 | 0 | 0 |  |  | 8 | 8 | |
| 2 | 11 | 41814 | 3 | 2 | 1 | 3 | 3 | 1 | 1 | 3 | 3 | 12 | 12 | |
| 2 | 12 | 241893 | 2 | 2 | 2 | 2 | 3 | 1 | 1 | 2 | 3 | 12 | 12 | |

Supplementary table. Number of piglets by treatment by sow according to the phase of the experiment.
